# A comparison of peer and faculty narrative feedback on medical student oral research presentations

**DOI:** 10.5116/ijme.5f64.690b

**Published:** 2020-09-30

**Authors:** Tracey A.H. Taylor, Stephanie M. Swanberg

**Affiliations:** 1 Department of Foundational Medical Studies, Oakland University William Beaumont School of Medicine, Rochester, Michigan, USA

**Keywords:** Formative feedback, medical students, oral presentations, research training curricula, undergraduate medical education

## Abstract

**Objectives:**

The purpose of this project was to evaluate and improve the oral presentation assessment component of a required research training curriculum at an undergraduate medical school by analyzing the quantity, quality, and variety of peer and faculty feedback on medical student oral research presentations.

**Methods:**

We conducted a program evaluation of oral presentation assessments during the 2016 and 2017 academic years. Second-year medical students (n=225) provided oral presentations of their research and received narrative feedback from peers and faculty. All comments were inductively coded for themes and Chi-square testing compared faculty and peer feedback differences in quantity, quality, and variety, as well as changes in feedback between the initial and final presentations. Comparative analysis of student PowerPoint presentation files before and after receiving feedback was also conducted.

**Results:**

Over two years, 2,617 peer and 498 faculty comments were collected and categorized into ten themes, with the top three being: presentation skills, visual presentation, and content. Both peers and judges favored providing positive over improvement comments, with peers tending to give richer feedback, but judges more diverse feedback. Nearly all presenters made some change from the initial to final presentations based on feedback.

**Conclusions:**

Data from this analysis was used to restructure the oral presentation requirement for the students. Both peer and faculty formative feedback can contribute to developing medical student competence in providing feedback and delivering oral presentations. Future studies could assess student perceptions of this assessment to determine its value in developing communication skills.

## Introduction

The development of effective communication skills, including providing feedback and giving oral presentations, tends to be challenging for students to master. Many health professions education accreditation bodies worldwide require students to be involved in research and to receive timely formative feedback. Research curricula provide an ideal opportunity for students to practice oral presentations and accrediting bodies in the United States and Canada, including medicine, nursing, physical therapy, occupational therapy, and nutrition, require or encourage student involvement in research (Table 1).^1^^-^^5^ Furthermore, accreditation standards for medicine, physical therapy, and occupational therapy education programs in the United States and Canada require that students receive formative feedback. For example, the Liaison Committee on Medical Education (LCME) requires that all medical students receive formal formative feedback in each course or clerkship (Standard 9.7).^5^ The Commission on Accreditation in Physical Therapy Education (CAPTE) requires that physical therapy students receive supervision and feedback during their clinical education (Standard 4J)^4^ and the Accreditation Council for Occupational Therapy Education (ACOTE) requires that all occupational therapy students are evaluated and provided feedback in a timely fashion (Standard A.3.5).^2^ Previous studies have found that timely formative feedback on oral presentations has been shown to improve student competence in oral presentations.^6^^-^^9^

**Table 1:** Overview of accreditation standards in various health care professions relevant to student participation in research

Discipline	Accreditation Body	Standards Document	Specific Standard(s) or Requirement(s)
Medicine (Undergraduate Medical Education or UGME)	Liaison Committee on Medical Education (LCME)	Functions and Structure of a Medical School (effective 2020 – 2021)	3.2 (Community of Scholars/Research Opportunities) – “A medical education program is conducted in an environment that fosters the intellectual challenge and spirit of inquiry appropriate to a community of scholars and provides sufficient opportunities, encouragement, and support for medical student participation in the research and other scholarly activities of its faculty.” 7.3 (Scientific Method/Clinical/Translational Research) – “The faculty of a medical school ensure that the medical curriculum includes instruction in the scientific method and in the basic scientific and ethical principles of clinical and translational research, including the ways in which such research is conducted, evaluated, explained to patients, and applied to patient care.”
Nursing	American Association of Colleges of Nursing (AACN)	The Essentials of Baccalaureate Education for Professional Nursing Practice	Essential III (Scholarship for Evidence-Based Practice) states that “dissemination is a critical element of scholarly practice; baccalaureate graduates are prepared to share evidence of best practices with the interprofessional team.”
Nutrition	Accreditation Council for Education in Nutrition and Dietetics (ACEND)	Accreditation Standards for Nutrition and Dietetics Coordinated Programs (effective July 1, 2018)	Standard 5.1 (Curriculum and Learning Activities) states that a program’s curriculum must include: “Research methodology, interpretation of research literature and integration of research principles into evidence-based practice.”
Occupational Therapy	Accreditation Council for Occupational Therapy Education (ACOTE)	2018 Accreditation Council for Occupational Therapy Education Standards and Interpretive Guide (effective July 31, 2020)	A.5.2 (Curriculum – Preparation and Application of In-Depth Knowledge) Students at the Baccalaureate and Doctoral levels are expected to apply in-depth knowledge in a variety of areas including research skills in the conduct of a project for their degree. B.6.1 (Scholarly Study) requires students at all levels from Associate Degree to Doctoral to at minimum understand scholarly activities and the contribution of literature to development of the profession. However, only the subset of students with a Doctoral degree have a requirement for original research.
Physical Therapy	Commission on Accreditation in Physical Therapy Education (CAPTE)	Standards and Required Elements for Accreditation of Physical Therapist Education Programs (revised December 7, 2017)	Standard 1B requires that programs have a documented goal that is based on PT research, however only the subset of students with a higher degree (Ph.D. or other doctoral degree) have a requirement for original research.

In courses, clerkships, or clinical education experiences where research is a component, formative peer feedback can be used to satisfy feedback standards, especially where feedback is otherwise difficult to provide. In the undergraduate literature, use of peer feedback on oral presentations has been reported in a variety of disciplines including the sciences^6^^,^^10^^,^^11^ business,^12^ engineering,^13^ and health sciences (nursing, nutrition, midwifery, and therapeutics).^7^^,^^14^^,^^15^ In undergraduate medical education (UGME), peer feedback has traditionally been used in the anatomy laboratory^16^^,^^17^ problem-based learning (PBL) activities,^18^^-^^20^team-based learning (TBL) activities^21^objective structured clinical examinations (OSCE),^22^^-^^24^and oral clinical case presentations.^8^^,^^25^ Formal education in developing and delivering oral research presentations provides a unique approach for improving student oral presentation competence. By learning to provide feedback, as well as doing oral presentations, medical students are introduced into the community of practice of medicine.

At Oakland University William Beaumont School of Medicine (OUWB), the Embark Program is a required, longitudinal scholarly concentration program spanning the four years of medical school.^26^ In this program, all medical students develop, conduct, and report on an independent, faculty-mentored research project meant to foster the development of four professional skills: team communication, research design, project operationalization, and time management.^26^ Following the first year of coursework in research design and project management, second-year medical students are taught research communication techniques including academic writing, drafting a scientific abstract,^27^ developing posters and oral presentations,^28^ and strategies for publishing. Specifically, students learn best practices for creating and delivering a five-minute oral presentation introducing their research and are assessed on their presentation skills by both peers and faculty.

In order to explore the natural teaching and learning environment and minimize researcher bias, no hypotheses were generated prior to initiation of this study. The aim of this project was to describe the quantity, quality, and variety of peer and faculty judge narrative feedback on medical student oral research presentations. An additional goal was to examine if and how students acted on this oral presentation feedback for a future research presentation in the same year. To our knowledge, previous studies have not reported analyzing peer feedback for medical student research presentations nor investigated differences in peer and faculty feedback on oral research presentations. Though focused on medical education, the findings of this project can be used to strategically strengthen the research curricula in other health professional education programs.

## Methods

A program evaluation of the oral research presentation component of the second-year Embark courses, “Techniques in Effective Scholarly Presentation” (winter semester) and “Embark Research Colloquium” (spring semester) was conducted.

### Participants

The Oakland University IRB determined that this project does not meet the definition of research under the purview of the IRB according to federal regulations. More specifically, this project was determined to be program evaluation.

**Figure 1:**
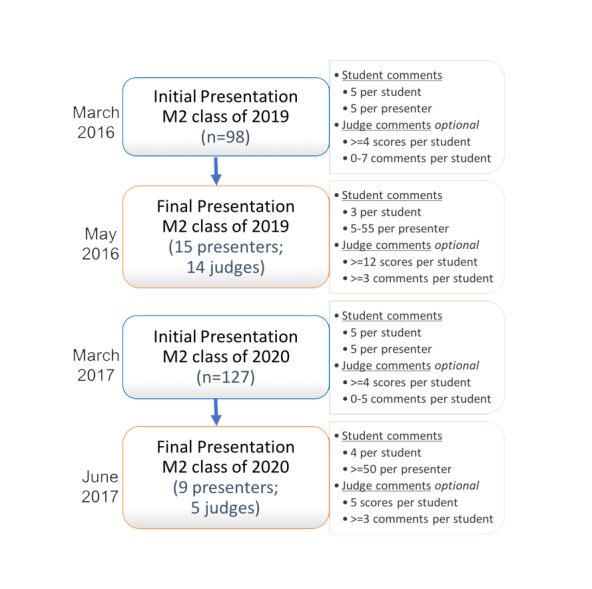
Flow diagram of medical student research oral presentations over two years

All second-year medical students (n=98 in 2016; n=127 in 2017) were required to present a mandatory “status update” oral presentation of their research during the winter semester (the “initial presentation”; see Figure 1). Students were provided with instruction as well as a PowerPoint template guide and divided into groups of 12-13 student presenters. They were allotted five minutes to present plus four minutes for questions from student and faculty attendees. A second requirement for each student was to provide narrative feedback to five assigned student peers during the presentations. All medical students are trained in providing narrative feedback to their peers during orientation in the first year of medical school, as well as assessed on the quality of their feedback during their first two years. The feedback consisted of three open-ended questions:

·       Briefly describe 1-2 things that were done well during this oral presentation (referred to as “done well”)

·       Briefly describe 1-2 things that could be improved in this oral presentation, and describe how improvement could be achieved (referred to as “improve”)

·       Any additional comments can be written here (optional)

In addition, each presentation was judged by a panel of at least four faculty judges. Judges were provided with a scoring rubric and offered optional training but were neither instructed in nor specifically encouraged to provide narrative feedback. All judges scored each presentation using the rubric and could opt to give narrative feedback.

The highest ranked students (n=15 in 2016; n=9 in 2017), as determined by normalized and pooled judge scores, were invited to present their research a second time (termed the “final presentation”; Figure 1) in the spring semester course. As students were not considered evaluators, peer feedback was not used in selecting the final group of presenters. A key spring course requirement was to present a research presentation (if chosen) or to provide narrative feedback to three or four student peers using the same three questions. Each presentation was again allotted five minutes plus four minutes for questions. Student presentations were again ranked and scored by faculty judges (n=14 in 2016; n=5 in 2017) using the rubric and the top three students were awarded the “Dean’s Choice Award” along with a monetary prize.

### Data analysis

All data (peer feedback, judge feedback, and student PowerPoint files) collected were de-identified and randomly assigned a subject identification number prior to analysis. Many of the collected peer and judge feedback comments contained more than one “thought”, and so those were separated prior to analysis. Multiple methods (qualitative and quantitative) were used to analyze the data. Thematic analysis of each comment from peers and faculty over the two years was conducted using inductive coding.^29^ In this method, themes emerge through an iterative process of reading and re-reading the data. This open coding was carried out independently by the two authors, followed by review, discussion, and revision of identified themes. Following this, a coding template was created using the identified themes and all comments were coded independently. At that point, any differences in coding were discussed and resolved. In addition to the qualitative analysis, frequency counts for each theme were calculated by totaling the coded comments in each category. Chi-square testing was used to compare changes in theme frequency between the initial and final presentations and between faculty and peer feedback. A p-value of <0.05 was considered statistically significant but was modified to consider the effect of clustering of comments for each student, when applicable. All analysis was done in SAS 9.4 (SAS Institute Inc., Cary, NC, USA).

Comparative analysis of student PowerPoint files from the two courses (before and after receiving initial presentation feedback) was conducted using the files from students that presented in both courses. Specifically, any changes in the design of the PowerPoint was noted including text, font size or style, background style, colors, graphics, and the notes field.

## Results

### Quantity of feedback

Overall, 3,115 narrative comments were collected from peers (n=2,617) and judges (n=498) over two years. Ten themes emerged which were lettered from A-to-I and NM (“No Meat”: comments that did not contain substantial content such as “good job” and “great presentation.”). The themes that were most prevalent were Content (E) and Presentation Skills (B), at frequencies of 29.6% (n=921) and 26.4% (n=822), respectively. In 2016, comments regarding Presentation Skills (B) were most frequent at 31.3% (n=396) followed by Content (E) at 24.3% (n=308), whereas in 2017 it was reversed with Content (E) at 33.1% (n=613) and Presentation Skills (B) at 23% (n=426). Comments in Visual Presentation (C) were the third most prevalent in both years (19.3% in total; 16.9% (n=214) in 2016; 20.9% (n=387) in 2017). The least frequent theme was Progress on Project (G) at only 0.2% over both years (0.3% (n=4) in 2016; 0.1% (n=2) in 2017). When comparing peer to judge feedback over both years, peer comments greatly outweighed judge comments in sheer number (Table 2). In 2016, peers provided a total of 937 comments while judges provided 328. The difference increased in 2017 with peers giving 1,680 total comments and judges only 170.

### Variety of feedback

Of the 2,617 peer comments over two years, 65.5% (n=1,713) described aspects of the presentations done well, with 34.5% (n=904) describing improvement suggestions. The majority of done well and improve comments were in the same three categories in both the initial and final presentations in both years: Content (E), Presentation Skills (B), and Visual Presentation (C).

Of the 498 comments from judges over two years, 64.5% (n=321) described what students did well while 35.5% (n=177) focused on improvement. Unlike students, the most frequently commented on categories by judges shifted between the two years: Presentation Skills (B; 30.8%; n=101), Content (E; 20.1%; n=66), and Visual Presentation (C; 17.4%; n=57) in 2016, and Content (E; 33.5%; n=57), No Meat (NM; 20.6%; n=35), Visual Presentation (C; 14.7%; n=25) and Presentation Skills (B; 14.1%; n=24) in 2017.

**Table 2:** Coding of major themes and total frequency of presenter comments

Coding Category	2016		2017		Total %	p-value ^*^<0.05
	Peer	Judge	Peer	Judge		
A=Preparation/Confidence/Passion	77	27	90	7	6.5%	0.71
B=Presentation Skills	295	101	402	24	26.4	0.45
C=Visual Presentation	157	57	362	25	19.3	0.08
D=Knowledge of Topic	33	21	46	3	3.3	^*^0.04
E=Content	242	66	556	57	29.6	^*^0.01
F=Study Design	4	5	7	3	0.6	^*^<0.01
G=Progress on Project	2	2	2	0	0.2	0.25
H=Interest/Relevance to Field	44	11	43	6	3.3	0.92
I=Answering Questions	28	15	62	10	3.7	0.09
NM=No Meat	55	23	110	35	7.2	^*^<0.01
Total	937	328	1,680	170	100.0	
Total Comments	1,265		1,850			
	3,115					

Note: Peer and judge feedback comments are shown with the total percentages combined. *p-values compare peer and judge total comments over two years, where judges were statistically more likely than medical student peers to comment on Knowledge of Topic (D), Study Design (F), and to make comments described as “No Meat” or too general (NM); and peers were more likely than judges to comment on Content (E).

When reviewing all comments, peers and judges prioritized different categories when giving feedback. In 2016, judges commented significantly more than peers on the presenter’s Knowledge of Topic (D; 10% (n=54) of comments for judges versus 5.3% (n=33) for peers (χ^2^ (1, N = 830) = 5.52, p=0.03) and Answering Questions (I; 6.6% (n=14) for judges versus 4.2% (n=26) for peers) (χ^2^ (1, N = 830) = 2.03, p=0.02) when describing what was done well, while peers commented significantly more than judges on Content (E; 22.8% (n=141) for peers versus 15.2% (n=32) for judges) (χ^2^ (1, N = 830) = 5.53, p=0.0002). In 2017, the differences between peer and judge comment categories were even more pronounced. When discussing what was done well, judges provided significantly fewer comments than peers related to Presentation Skills (B; 14.5% (n=16) for judges versus 22.4% (n=245) for peers) (χ^2^ (1, N = 1204) = 3.63, p=0.04) and Content (E; 15.5% (n=17) for judges versus 30.6% (n=335) for peers) (χ^2^ (1, N = 1204) = 11.11, p=0.01). The only category in which judges provided significantly more feedback on what was done well was No Meat (NM) at 31.8% (n=35) of comments versus only 7.9% (n=86) of peers (χ^2^ (1, N = 1204) = 63.46, p=0.0001). When providing feedback on what could be improved, judges commented significantly more on Content (E) (χ^2 ^(1, N= 646) = 18.95, p=0.0001) with 66.7% (n=40) of comments versus only 37.7% (n=221) of peer comments and Study Design (F) with 3.3% (n=2) of comments compared to only 0.3% (n=2) of peer comments (χ^2^ (1, N = 646) = 7.92, p=0.02). However, peers focused significantly more on Presentation Skills (B) with 26.8% (n=157) of comments versus only 13.3% (n=8) of judge comments (χ^2^ (1, N = 646) = 5.18, p=0.01) and Visual Presentation (C) with 25.9% (n=152) of comments compared to only 10% (n=6) of judge comments (χ^2^ (1, N = 646) = 7.48, p=0.001). In general, judge feedback tended to be more diverse in scope providing comments in all categories. In fact, in both years, judges commented in all categories except for Progress on Project (G) related to what the presenters did well and Knowledge of Topic (D) for what they could improve.

### Comparison of initial and final presentation feedback

When comparing initial and final presentation feedback, interesting differences between judge and peer comments emerged. In relation to how categories significantly decreased over the two years, in the initial presentations in 2017, judge improve comments decreased for both Presentation Skills (B; 37.5% (n=6) to 4.5% (n=2); (χ^2^ (1, N=60) = 11.03, p=0.0001) and Visual Presentation (C; 18.8% (n=3) to 6.8% (n=3); (χ^2^ (1, N=60) = 1.86, p=0.03). The trend was similar in 2016 with a significant decrease (χ^2^ (1, N=117) = 2.80, p=0.01) in the number of judge improve comments related to Visual Presentation (C) from 26.4% (n=14) in the initial presentation to 14.1% (n=9) in the final presentation. In 2016, there was a significant decrease (χ^2^ (1, N = 211) = 3.31, p=0.05) in the number of No Meat (NM) comments from judges from the initial 13.5% (n=15) to final presentations 6% (n=6) related to what students did well. For students, there was no statistically significant decrease in feedback from the initial to the final presentations in any category.

In contrast, many categories saw an increase from the initial to the final presentations, most related to done well comments. Judge comments in 2017 on Content (E) significantly increased from 7% (n=4) to 24.5% (n=13) of comments (χ^2^ (1, N=110) = 6.45, p=0.01) when discussing aspects of the presentation done well, but, surprisingly, also increased for student improvement (31.3% (n=5) to 79.5% (n=35); (χ^2^ (1, N = 60)=12.32, p=0.0001). In terms of peer comments of presentation aspects done well during the first year, there were significantly more related to Interest/Relevance to the Field (H) during the final presentations with 8.9% (n=41) compared to only 1.9% (n=3) in the initial presentations (χ^2^ (1, N=619)=8.61, p=0.0001). In both years, students commented significantly more frequently that presenters answered questions well (I) in the final presentations with 5% (n=23) in 2016 and 5.4% (n=53) in 2017 of comments respectively when compared to the initial presentations with only 1.9% (n=3 in 2016; n=2 in 2017) for each year (χ^2^ (1, N = 619) = 2.74, p=0.01 in 2016; χ^2^ (1, N=1094)=2.42, p=0.03 in 2017). No Meat (NM) comments from both peers and judges related to presentation aspects that presenters did well were overall more frequent in the final presentations: during the second year, there were 8.3% (n=82) NM peer comments in the final presentations compared to only 3.8% (n=4) (χ^2^ (1, N = 1094)=2.71, p=0.01) in the initial presentations and 39.6% (n=21) of the final presentation judge comments compared to 24.6% (n=14) of initial presentation comments (χ^2^ (1, N = 110) = 2.87, p=0.03).

### Comparison of presentation file changes by individual students

In addition to reviewing narrative feedback, individual student presentation PowerPoint files were analyzed. Because analysis relied on the collection of written feedback comments from both student peers and faculty judges, it was possible to make qualitative individual student comparisons between the initial and final presentations. This analysis yielded a plethora of data for the 24 students.

Comparison of PowerPoint file changes made by students from the initial to the final presentations in each year revealed some interesting observations. In 2016, three of the 15 (20%) student presenters chose to make no changes to their presentation files; four students (26.7%) made small modifications (such as changing one single word in the entire file); and six (40%) made moderate changes (such as one large change that affected multiple slides in addition to modifying wording on two others). Two of the 15 (13.3%) students made large changes such as changing the background, changing the slide order, adding graphics, removing significant text, and/or adding emphasis to aspects of the slides. In 2017, only one of the nine students (11.1%) did not make changes to the presentation file (decrease over the previous year); four students (44.4%) made small changes (increase over the previous year); and two students each made moderate changes (22.2%; decrease over the previous year) and large changes (22.2%; increase over the previous year).

## Discussion

The major finding of this study was that formative feedback from peers and faculty judges on oral presentation skills differs substantially in quantity, quality, and variety. Additionally, fewer students than expected utilized this feedback to improve their presentations for a future oral presentation. As this data was collected from an active course rather than a controlled study environment, judge and student feedback may more accurately reflect experiences and observations of the natural teaching and learning environment. To the authors’ knowledge, this is the first evaluation that analyses trends in peer and faculty feedback of medical student oral research presentations over time.

### Impact of instruction & feedback on oral presentation skills

Several studies have shown that instruction in oral presentation skills followed by practice and feedback improve students’ ability to design and deliver effective presentations in both medical education^8^^,^^9^^,^^25^^,^^30^ and higher education settings.^7^^,^^31^ For example, one study designed an online clinical reasoning curriculum for second-year medical students, which included modules on oral presentation skills. The authors assessed the intervention groups’ presentations over three time points and their scores improved while the control group scores declined.^8^ Another study assessed the use of formative feedback from faculty on third-year medical student oral clinical case presentations during a pediatric clerkship and found similar improvements.^30^ This previous research supports the importance of integrating required instruction in oral presentation development and delivery into the OUWB Embark Program. The four professional skills developed through Embark: team communication, research design, project operationalization, and time management are essential lifelong skills that medical students can translate to their future clinical, academic, and research practices.^26^

### Multi-source feedback

It is important to teach medical students how to design and deliver oral presentations as well as to include various sources of assessment when evaluating student performance. The positive impact of multiple sources of feedback specifically related to oral presentation skills has been well studied in higher education literature.^24^^,^^31^^-^^34^Our analysis discovered substantial differences in the quantity, variety, and quality of narrative feedback by peers and faculty.

### Quantity of feedback

The sheer quantity of narrative comments given by peers and faculty judges varied dramatically with peers providing 2,617 comments over the two years and judges only providing 498 comments. This difference is primarily a result of how the feedback process was structured within the course (Figure 1). For the initial presentations, each presenter received feedback from five peers and four or more judges. For the final research presentations in 2016, students were required to select at least three of the 15 finalists in which to provide narrative feedback. Therefore, all 83 non-presenting students provided feedback with only fourteen judges providing scores for the 15 finalists. As students tended to provide feedback to the same students (the earlier presentations in the colloquium), the process was modified the following year. In 2017, students were assigned four peers to ensure that all student presenters received equal feedback during the final presentations. Judge narrative feedback was optional and each student received between zero and seven judge comments. Over the two years, the number of judges present at both the initial and final presentation sessions varied. The difference in quantity of feedback could dramatically affect the quality, as discussed later.

### Variety of feedback

In regard to type of feedback provided, our analysis found that both faculty and peers mostly commented in the same three categories: Visual Presentation (C), Content (E), and Presentation Skills (B) (Table 2). However, faculty feedback was much more varied, leading to the development of new themes in our analysis: Knowledge of Topic (D), Study Design (F), Project Progress (G), Relevance to the Field (H), and Answering Questions (I). This difference in breadth of feedback between peers and faculty may simply be due to faculty’s previous experience in conducting research and delivering oral presentations. Indeed, faculty’s tacit knowledge and experience has been identified in previous studies as a major factor in differences between self, peer, and/or faculty feedback.^32^^,^^33^ As Magin and Helmore^33^ said:

“teachers are more experienced, more expert, and are less likely to be biased in their judgements.”

The variety of themes identified in our analysis (Table 2) are somewhat different from previously published studies. Many oral presentation rubrics published in the literature focus ondelivery, presentation content, and visual presentation.^7^^,^^12^^,^^32^^,^^35^ For example, De Grez and colleagues^32^ divided oral presentation elements into two categories: delivery (eye contact, vocal delivery, enthusiasm, interaction with audience, body language) and content (quality of introduction, structure, conclusion, professionalism). In these areas, our study found similar trends for peer and judge feedback. Previous medical education literature has focused on students’ ability to deliver an oral clinical case presentation, where elements related to gathering a patient history and physical exam findings are included.^25^^,^^36^^,^^37^ But the themes identified through our analysis reflect the elements unique to research presentations: Study Design (F), Project Progress (G), and Relevance to the Field (H), all important concepts for students in health sciences fields to learn as part of research training curricula.

### Quality of feedback

Our analysis also revealed that the quality of student and faculty feedback differed substantially. Both peers and judges tended to be positive, providing twice as many done well comments as improve comments. In addition, judge feedback shifted to be more general from 2016 to 2017 with the number of done well comments in the No Meat (NM) category rising from 10.0% (n=21) in 2016 to a surprising 31.8% (n=35) in 2017 (χ^2^ (1, N=321)=24.00, p=0.0001). This differs from previously reported literature where faculty tend to judge students more critically than peers in oral presentation skills.^31^^-^^33^In the study by De Grez and colleagues,^32^ faculty scores were significantly lower than both peer- and self-assessments.^32^ However, in a study by Wettergreen and colleagues,^34^ comparisons of faculty and self-assessment scores of pharmacy students’ performance in clinical case discussions were found to be similar, which more closely mirrors our findings. Some of this difference may be due to the narrative prompts given to students but not to judges in our process. Students were required to comment on one thing the presenter did well and one area for improvement whereas judges were given an optional comment box with no prompt. Furthermore, our medical students are trained and assessed in providing both positive and constructive narrative feedback to their peers based on recommendations by Michaelsen and Schultheiss^38^ as part of TBL orientation in the first year of medical school. For our oral presentations, judges were offered training for the scoring rubric, but were neither instructed in nor specifically encouraged to provide narrative feedback to the student presenters. This could potentially be remedied in the future if judges are given the same two prompts and required to provide narrative comments to students in addition to quantitative scores using the rubric.

### Trends in feedback between initial & final presentations

In comparing student presentation skills over time, our analysis shows that both peer and judge comments shifted between the initial and final presentations implying that the feedback on initial presentations was utilized by students to alter their final presentations. For example, in 2016, there were significantly more peer done well comments in Interest/Relevance to the Field (H) during the final presentations (8.9%; n=41) when compared with the initial presentations (1.9%; n=3) (χ^2^ (1, N= 619) = 8.61, p=0.0001), suggesting that revisions to the presentations spurred an increased interest of the research topic. Students also commented significantly more frequently that their peers answered questions well (I) in the final presentations (5%; n=23) when compared to the initial presentations (1.9%;n=3) (χ^2^ (1,N=619)=2.74, p=0.01), suggesting that student presenters answered questions more effectively with practice and preparation. That same year, judge improve comments related to Visual Presentation (C) saw a significant decrease (χ^2^ (1, N=117) = 2.80, p=0.01) from 26.4% (n=14) in the initial to 14.1% (n=9) in the final presentation. These findings suggest that students used both peer and judge formative feedback to revise the content or delivery from one presentation to the next. Indeed, two representative students did make changes to their presentations suggested by the feedback. It is interesting that even though the second representative student made the changes suggested by peers (darker background to slides), in the final presentation many peers commented that they would have preferred the initial color scheme (lighter background to slides). It was surprising that more students did not choose to utilize the feedback for substantial changes to their PowerPoints. This could be due to the fact that dedicated preparation time for medical school licensure examinations was scheduled immediately prior to the final presentations.

### Limitations and future directions

This study has some limitations. First, as the original intent of this analysis was to examine the differences between peer and faculty feedback in oral presentation performance as part of the course, this was not designed as a research study. It is difficult to directly assess the impact, value, and student use of feedback in developing and delivering oral presentations. Our assumptions of value are based on student changes to their presentations and trends in peer and faculty feedback between the initial and final presentations. Future research studies could employ other methods of assessment such as critically analyzing video recordings of student presentations or assessing changes in the quantitative scores of judges from the initial to the final student presentations. Other studies could include surveying medical students to assess changes in attitudes and perceived value of the activity prior to and following the oral presentation component.

Second, this project likely includes selection bias as only feedback from the top students were analyzed rather than comments for the entire class. However, as no new themes emerged after analyzing the data over two years, we believe the data are representative.

Though not a limitation, direct comparisons of student and judge comments were a challenge based on several confounding factors. As mentioned previously, because the oral presentation activity was integrated into the curriculum, students were required to provide one positive comment and one area for improvement to a defined number of peers as a course requirement. Judges, on the other hand, served as volunteers and expectations regarding narrative feedback were not explicit. The scoring form included quantitative items with an optional and open-ended comments box for judges to provide feedback. In addition, because medical students were formally trained in giving narrative feedback for TBL and judges were not trained or coached, this may have affected the quantity and quality of peer versus judge feedback.

## Conclusions

Our program evaluation analysis led to several changes in the oral presentation component of the courses from 2016 to 2017 including contributing to the development of a new rubric based on themes identified, revising the requirements for peer feedback to more evenly distribute comments across the student finalists, and encouraging judges to provide narrative feedback in 2017. Developing effective communication skills, whether in the context of clinical care or for research purposes, is an essential skill for all medical students as they enter the community of practice of medicine. Key components of communication are the abilities to give feedback as well as deliver oral presentations, though medical schools have focused much of their curricula on clinical case presentation skills. Students who learn how to effectively conduct and communicate research should be better prepared to practice evidence-based medicine in their future medical practice. The educational method described herein could be easily adapted and implemented at other health care educational institutions worldwide that include a scholarly concentration or research component in the curriculum. This analysis provides the groundwork for further investigation of the impact of both peer and faculty feedback on developing medical student oral presentation competence.

### Acknowledgements

The authors wish to thank Patrick Karabon, MS, for his assistance with statistical analysis. We wish to thank Drs. Stefanie Attardi, PhD and Stephen Loftus, PhD for critically reviewing the manuscript. We also wish to thank Keith Engwall, MS LIS, for his role as the chair of the M2 Embark Presentation Committee and Stephen Loftus, PhD for his role as Embark co-course director for the second-year courses. We are grateful to Dwayne Baxa, PhD and Kara Sawarynski, PhD for their leadership as the Directors of the Embark Program, and Julie Strong who serves as the Embark course coordinator.

### Conflict of Interest

The authors declare that they have no conflict of interest.
